# Supplementary material for: DNA Methylation and Alternative Splicing Safeguard Genome and Transcriptome After a Retrotransposition Burst in Arabidopsis thaliana
Source: Int J Mol Sci. 2025 May 17;26(10):4816. doi: 10.3390/ijms26104816 (PMC12112155; doi:10.3390/ijms26104816)
Supplement: Supplementary file 1 [file ijms-26-04816-s001.zip › Supplementary figures.pdf]

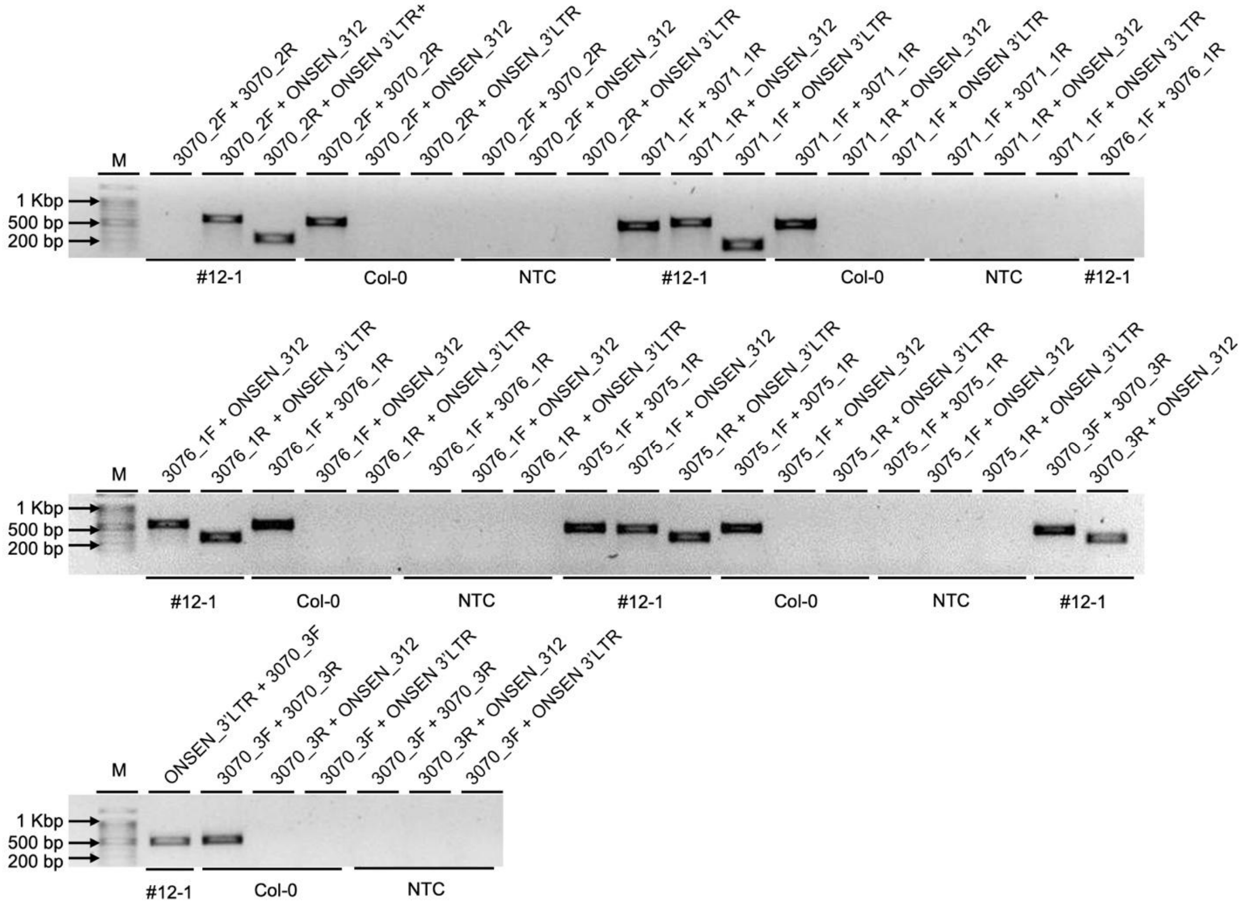

Figure S1. **PCR validation of five novel *ONSEN* insertions in plant #12-1.** M - molecular weight marker; #12-1 - total DNA from plant #12-1; Col-0 - total DNA from wild-type Arabidopsis thaliana Col-0; NTC - no template control. Primers used are listed in **Table S9**.

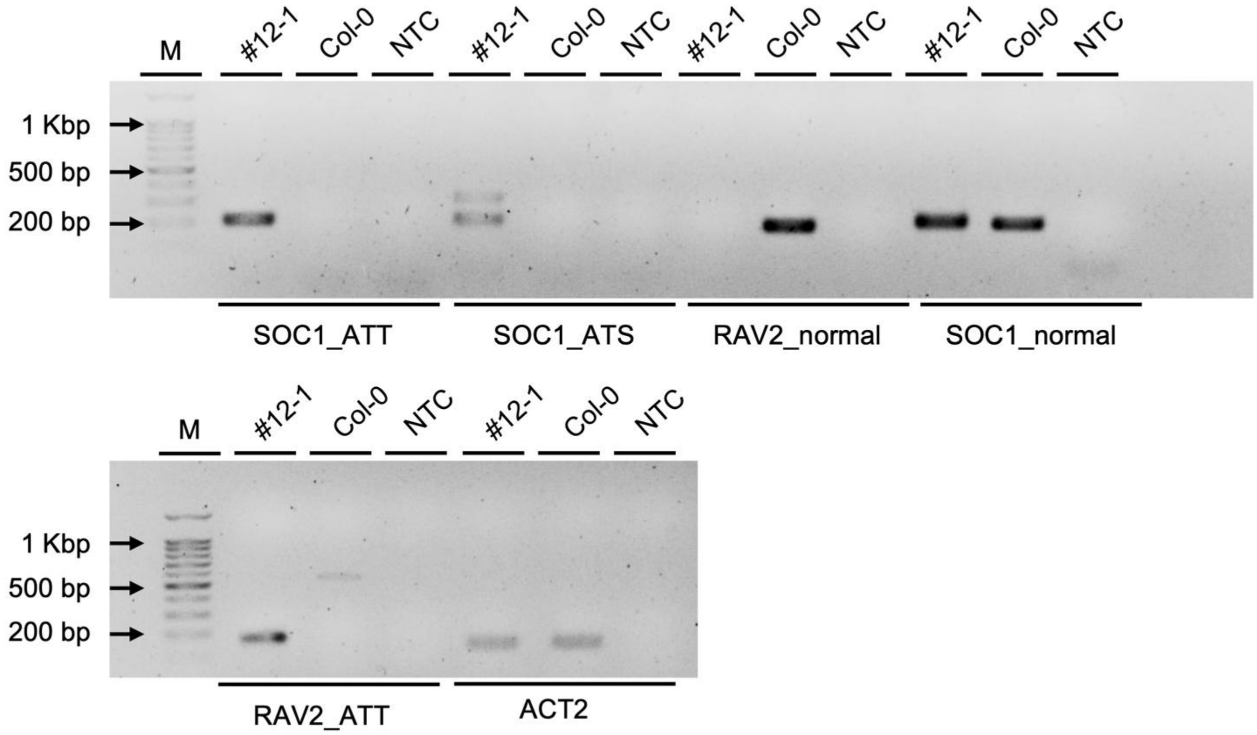

Figure S2. **PCR validation of alternative splicing events in the *SOC1* and *RAV2* genes harboring novel *ONSEN* insertions.** M - molecular weight marker; #12-1 - cDNA from plant #12-1; Col-0 - cDNA from wild-type *Arabidopsis thaliana* Col-0; NTC - no template control. in plant #12-1. Primers used are listed in **Table S9**.

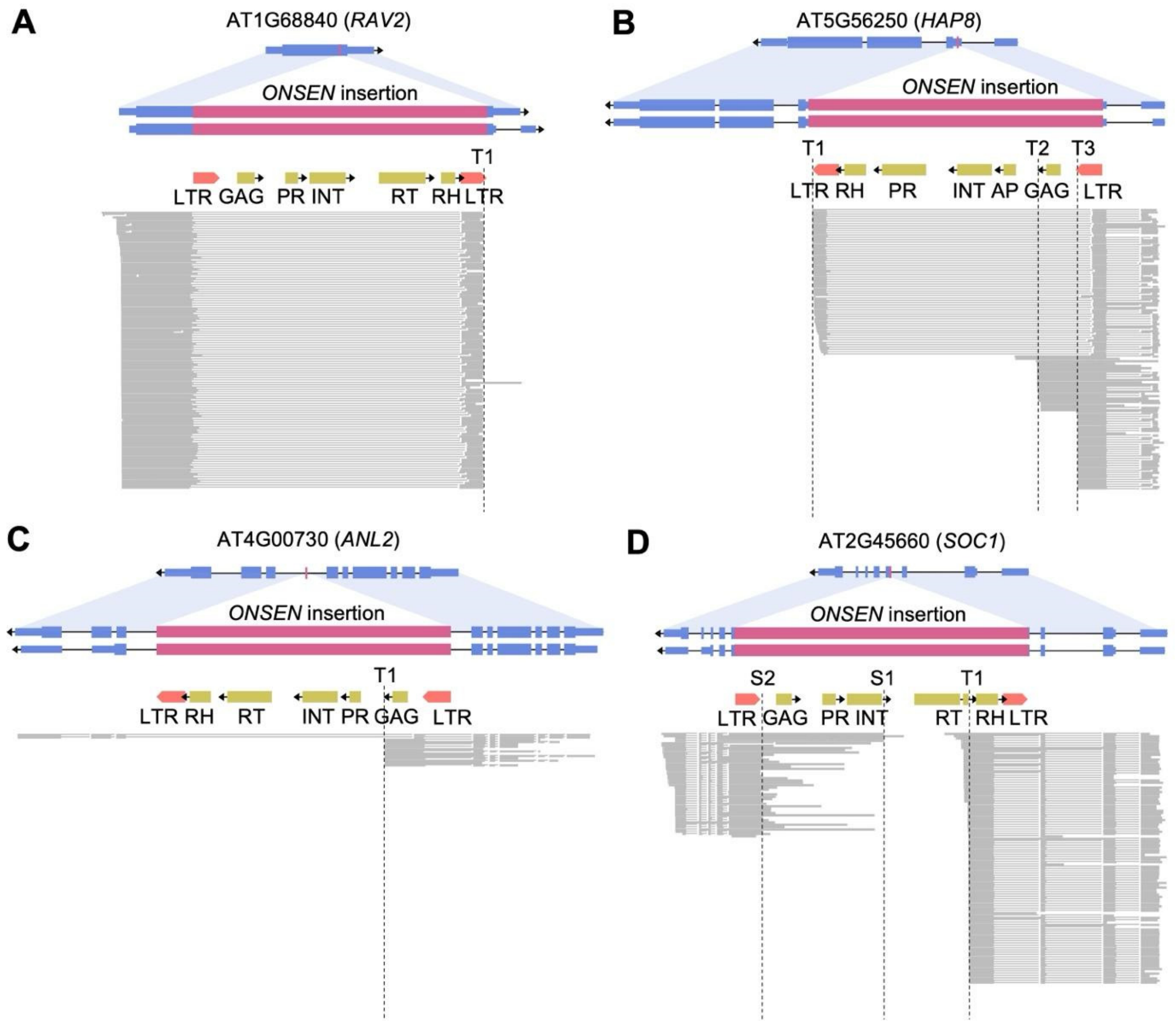

Figure S3. **Gene-TE isoforms for genes with *ONSEN* TEIs in #12-1**. (**A**) TTS in R region of LTR of *RAV2* gene. (**B**) Multiple TSSs in GAG-RT sequence space and TTS in RT-RH sequence space of *SOC1* gene. (**C**) Multiple TTS in R region of LTR and GAG-PR sequence space in *HAP8* gene; (D) TTS in GAG-PR sequence space in *ANL2* gene. Abbreviations of TE domains: LTR – Long Terminal Repeat; GAG – Group-specific Antigen; PR – Protease; INT – Integrase; RT – Reverse Transcriptase; RH – RNase H.

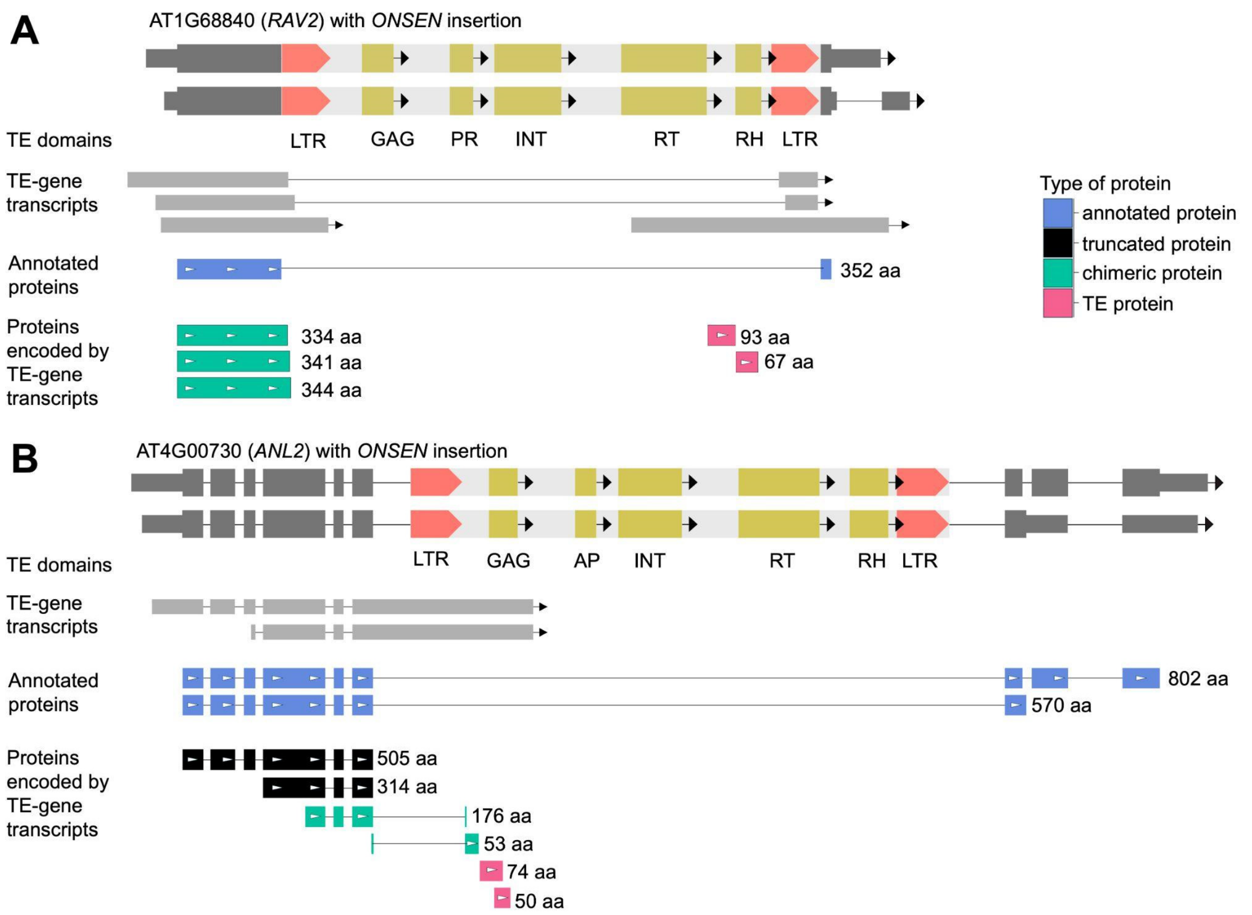

Figure S4. Open reading frames (ORFs) prediction within TE-gene transcript isoforms. (**A**) AT1G68840 (*RAV2*) TE-gene transcript analysis. (**B**) AT4G00730 (*ANL2*) TE-gene transcript analysis. Transcript isoforms were reconstructed using StringTie2 v.2.2.1, with ORFs predicted by NCBI ORFfinder tool. Abbreviations of TE domains: LTR – Long Terminal Repeat; GAG – Group-specific Antigen; PR – Protease; INT – Integrase; RT – Reverse Transcriptase; RH – RNase H.

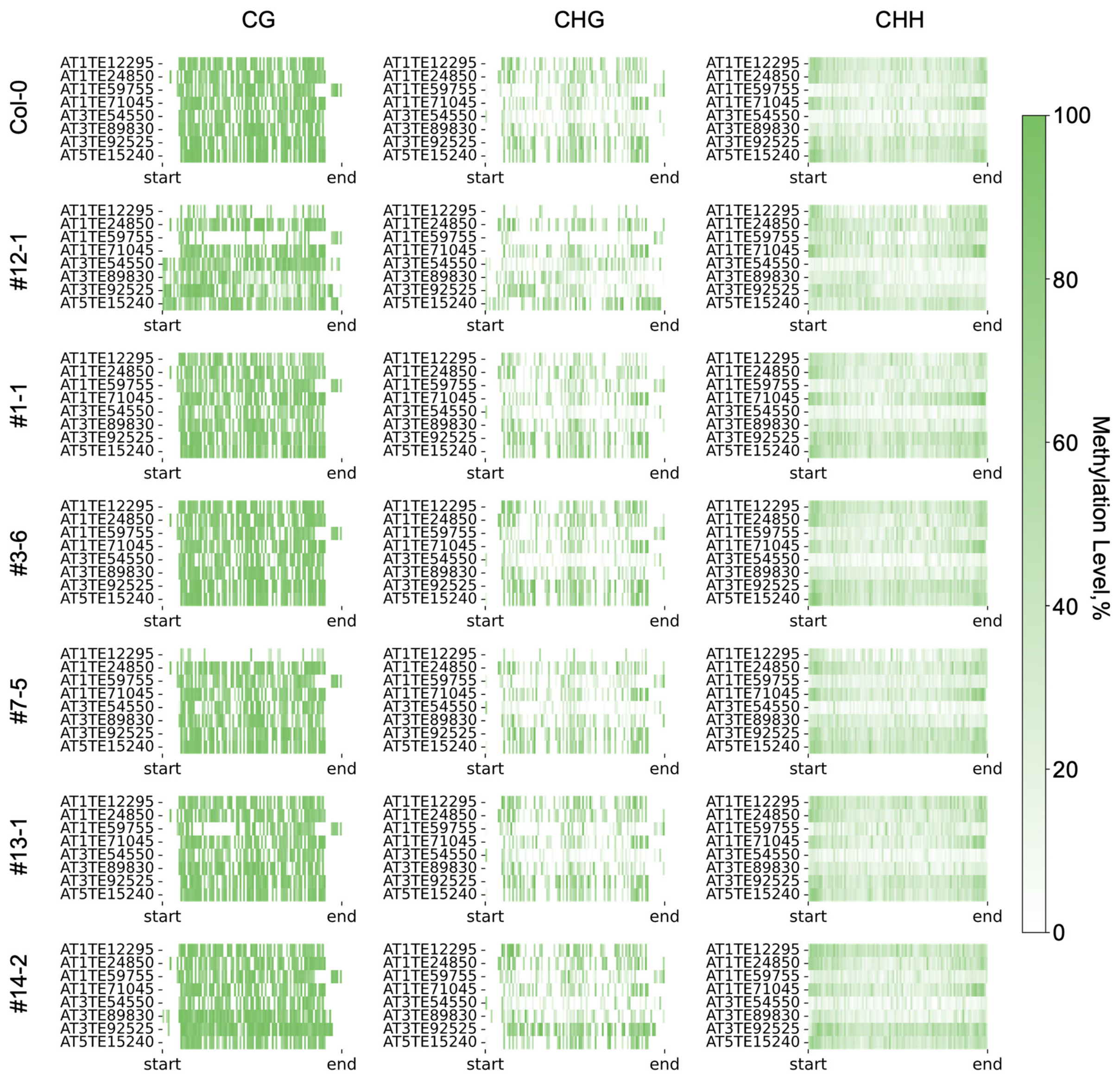

Figure S5. Original ONSEN copies body methylation.

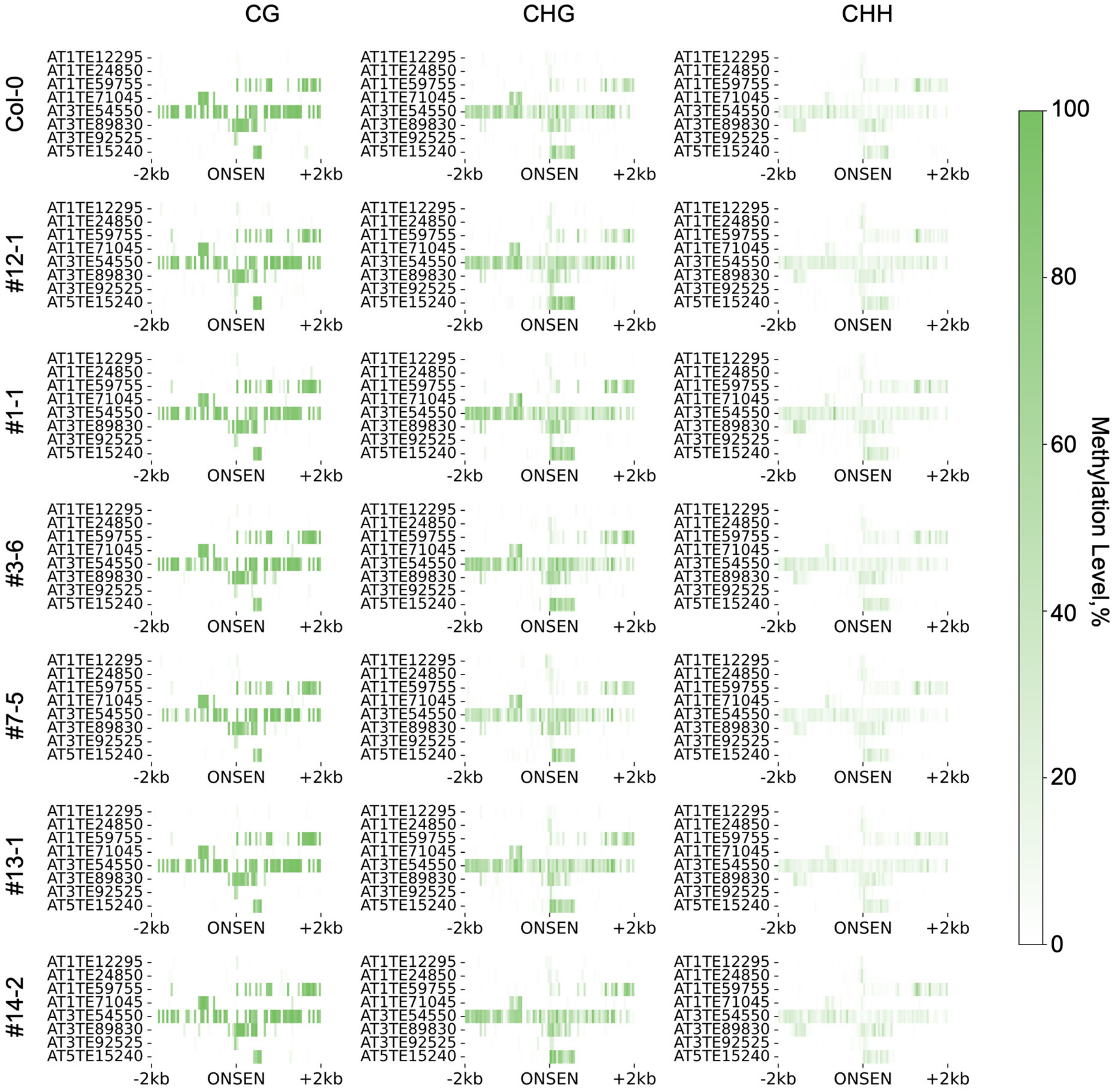

Figure S6. Original *ONSEN* copies flanking regions methylation.

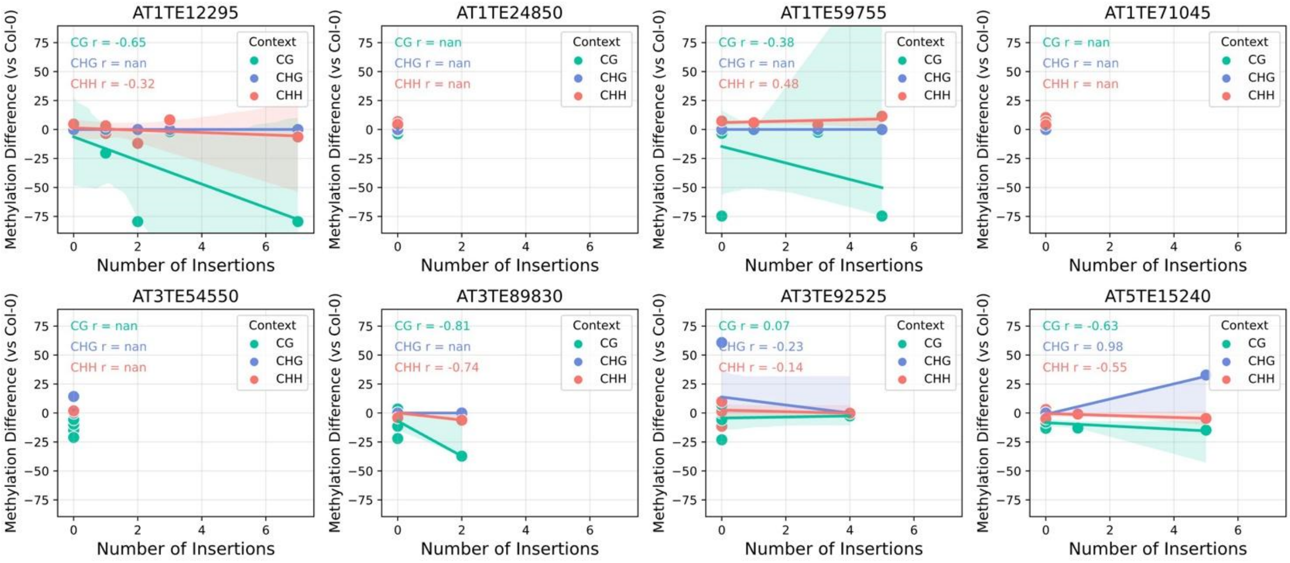

Figure S7. Correlation between median methylation level difference (M1 samples vs Col-0, %) and number of insertions produced by each *ONSEN*. Dots represent samples with corresponding ONSEN insertions. Line represents linear regression model fit, shadowed area is confidence interval, r is Pearson correlation coefficient.

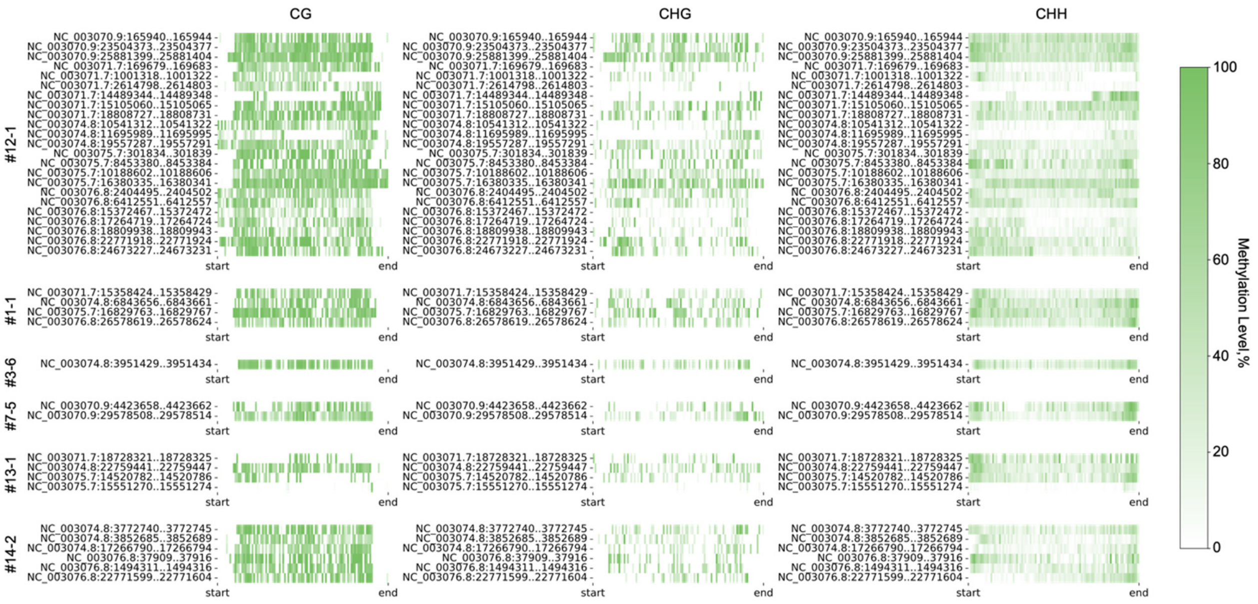

Figure S8. Methylation levels in selected TEIs. Mean binned (100 bins) combined methylation levels from two biological replicates are shown (except for #14-2).

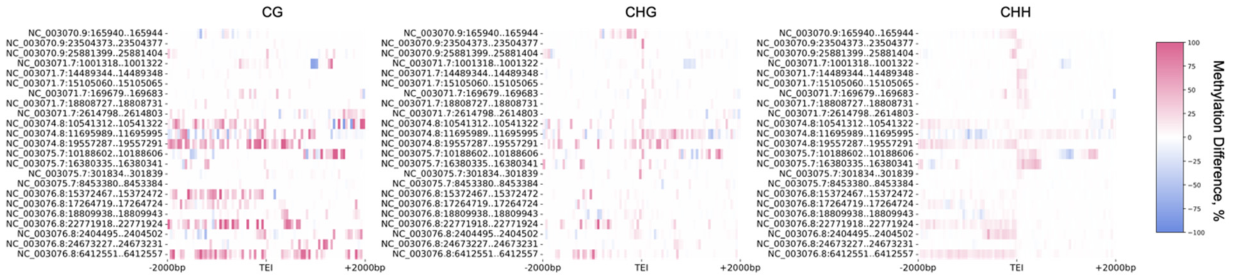

Figure S9. Methylation level difference between #12-1 TEI flanking regions and corresponding positions in Col-0. Mean binned (50 bp) combined methylation levels from two biological replicates are shown.
